# Computational Analysis of the Binding Specificities of PH Domains

**DOI:** 10.1155/2015/792904

**Published:** 2015-12-31

**Authors:** Zhi Jiang, Zhongjie Liang, Bairong Shen, Guang Hu

**Affiliations:** ^1^Center for Systems Biology, Soochow University, Suzhou 215006, China; ^2^Department of Biochemistry and Molecular Biology, School of Medicine, Soochow University, Suzhou 215123, China

## Abstract

Pleckstrin homology (PH) domains share low sequence identities but extremely conserved structures. They have been found in many proteins for cellular signal-dependent membrane targeting by binding inositol phosphates to perform different physiological functions. In order to understand the sequence-structure relationship and binding specificities of PH domains, quantum mechanical (QM) calculations and sequence-based combined with structure-based binding analysis were employed in our research. In the structural aspect, the binding specificities were shown to correlate with the hydropathy characteristics of PH domains and electrostatic properties of the bound inositol phosphates. By comparing these structure properties with sequence-based profiles of physicochemical properties, PH domains can be classified into four functional subgroups according to their binding specificities and affinities to inositol phosphates. The method not only provides a simple and practical paradigm to predict binding specificities for functional genomic research but also gives new insight into the understanding of the basis of diseases with respect to PH domain structures.

## 1. Introduction

PH (pleckstrin homology) domains, consisting of about 100–120 amino acid residues, are found in a wide range of proteins involved in intracellular signalling or as constituents of the cytoskeleton [1–4]. Although their sequences bear very low similarity, all the determined three-dimensional structures have seven *β*-strands forming two perpendicular anti-parallel-sheets and one C-terminal *α*-helix [5]. PH domains bind either plasma-membrane phosphoinositides or cytosolic inositol phosphates with few exceptions. The specific binding of PH domains to different inositol phosphates is important for the signal-dependent membrane targeting [6–8]. To date, the most studied inositol phosphates include inositol 1,3,4-triphosphate (Ins(1,3,4)-P_3_), inositol (1,4,5)-trisphosphate (Ins(1,4,5)-P_3_), and inositol 1,3,4,5-tetrakisphosphate (Ins(1,3,4,5)P_4_). Some PH domains have been shown to interact also with protein kinase C or heterotrimeric G proteins [9, 10] in signalling pathways. In addition, PH domains are increasingly found to be connected to human disorders. For examples, Bruton tyrosine kinase (Btk) is involved in X-linked agammaglobulinemia (XLA) [11, 12], an immunodeficiency. FGD1 protein and AKT1 are connected to Aarskog-Scott syndrome (ASS) [13] cancer [14, 15], respectively.

In the present work, we will focus on eleven well-known PH domains whose three-dimensional structures have been determined. Five out of these PH domains bind inositol phosphates with different specificities [16–21]. In addition to evolution, PH domains have been divided into four functional subclasses according to binding affinities and specificities [2] (Table 1). In summary, PH domains in Group 3 bind their preferred ligands with similar affinities to those in Group 1, whereas PH domains in Group 2 have 4–8-fold weaker binding affinities. In addition, PH domains in Group 4 have low affinity and less specificity. Concerning the three inositol phosphates, the affinity of Ins(1,4,5)P_3_ to its preferred PH domains is generally lower than those of Ins(1,3,4,5)P_4_ and Ins(1,3,4)P_3_ [22, 23]. To understand the sequence-structure relationship of the four groups of PH domains, we have investigated a number of sequence profiles to characterize their physicochemical properties [24]. More recently, molecular dynamics (MD) simulations were performed to study structural and binding affinity of functional mutations of Btk PH domains [25] and the role of membrane penetration and electrostatics in the interaction between GRP1 PH domain and PI(3,4,5)P_3_ [26, 27].

Despite the large body of PH domain literature, some problems are urgently to be resolved, such as the following: (1) Why the binding affinity of Ins(1,4,5)P_3_ to its preferred PH domains is weaker than those of the other two inositol phosphates? (2) What factors determine the binding specificities of PH domains for different inositol phosphates? (3) Considering the fact that PH domains have low sequence identities but highly conserved structures, is there any intrinsic relationship between the binding specificities and sequence profiles of physicochemical properties? To these aims, a systematic comparison of the sequence-structure-function relationship is needed for further research of the functions of PH domains. In this paper, we first calculated the properties of both the inositol phosphates and PH domains, and then the binding specificities and affinities of PH domains were analyzed from both the structural and sequence aspects.

## 2. Materials and Methods

### 2.1. QM Calculations

The geometries of the three inositol phosphates, Ins(1,3,4)P_3_, Ins(1,4,5)P_3_, and Ins(1,3,4,5)P_4_, were optimized at the density functional theory (DFT) of B3LYP/6-31+G(d,p) level [28] and the equilibrium structures were verified with calculations of frequencies. The single point energies and the electronic properties of the optimized inositol phosphates were calculated at the B3LYP level with the same basis sets. All the calculations were performed with Gaussian03 [29]. The initial conformations of Ins(1,4,5)P_3_ and Ins(1,3,4,5)P_4_ were extracted from PDB entries 1B55 and 1MAI, respectively. The initial conformation of Ins(1,3,4)P_3_ was modified from Ins(1,3,4,5)P_4_.

### 2.2. Sequence Analysis

**Figure 1:**
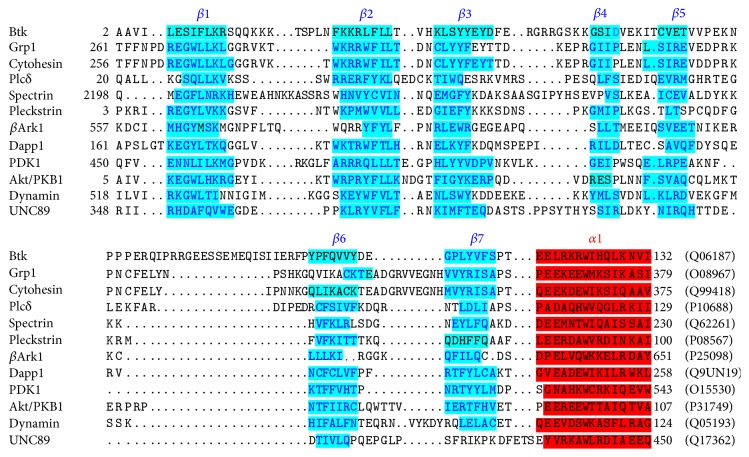
Three-dimensional structure-based sequence alignment of PH domains by the method of hidden Markov model. The SwissPort accession numbers are shown at the end of the sequences.

Because the sequence similarities of PH domains are limited (average identity is 16%), the multiple sequence alignment could not be obtained with the general alignment programs. The sequence alignment of the selected PH domains was retrieved from the protein family (Pfam) database, which is created based on hidden Markov model [30]. The sequence alignment was further modified based on structure-based alignment and shown in Figure 1. The PH domains were selected to represent the different functional subgroups including Btk (PDB code: 1BTK, 1B55), Grp1 (1FGZ, 1FGY, 1FHW, 1FHX), Plc-*δ* (1MAI), spectrin (1BTN, 1MPH, 1DRO), pleckstrin (1PLS), *β*-Ark (1BAK), Dapp1 (1FB8, 1FAO), dynamin (1DYN), and UNC-89 (1FHO). MEGA6 was used to construct phylogenetic tree of PH domains based on maximum composite likelihood method [31]. The profiles of physiochemical properties of PH domains, including flexibility, hydropathy, isotropic surface area, and electronic charge concentration, were calculated as previously described [24].

### 2.3. Structure and Binding Analysis

The electrostatic potentials of PH domains were calculated using a finite different solution to the nonlinear Poisson-Boltzmann equation [32]. The grid was 20 Å larger than the PH molecule containing 123 grid points in the longest dimension. The solute dielectric was set to 2. Solvent accessible surface areas (SASA) were calculated according to the algorithm of Lee and Richards [33], and a solvent radius of 1.4 Å was used for water. The fractions of residues exposed to solvent were calculated directly from the experimental structures and were subsequently used to generate profiles with the sliding window averaging technique to facilitate comparison to the predicted properties. In the case of NMR structures missing the average structure (1MPH, 1PLS, 1BAK, and 1FHO), the first structure in the entry was used. The detailed interatomic contacts for inositol phosphate to PH domains were investigated with the LIGIN program [34]. All the other structural analyses were performed with InsightII software of Accelrys, Inc.

## 3. Results and Discussion

**Figure 2:**
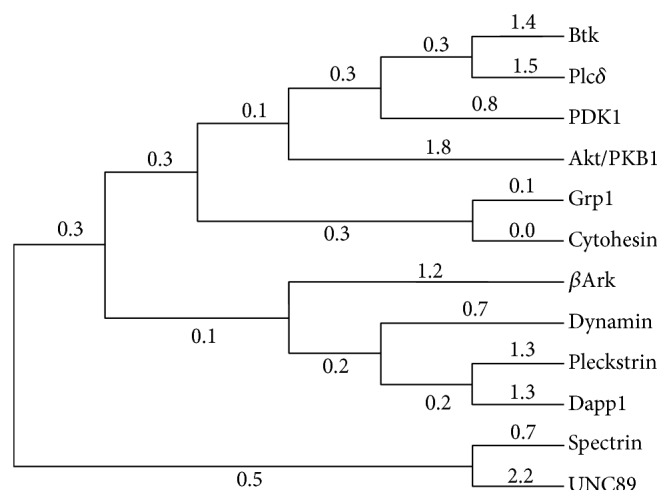
The phylogenetic tree for 12 PH domains. Bootstrap analysis was carried out using MCL approach, and bootstrap values are shown as scores of branches.

Binding PH domains have been identified in various species [35, 36], and a few reports have discussed the evolution of TFKs including PH domains [37]. Evolutionary relationship among different groups of PH domains is of interest to be compared with binding specificities. The phylogenetic tree for 12 PH domains (Figure 2) was constructed based on the sequence alignment. Apparently, four groups are not clearly classified in the phylogenetic tree. For example, Btk, Plc-*δ*, and PDK1, belonging to three different groups, have nearly phylogenetic relationship. This result may be attributed to the low sequence identities but extremely conserved structures in PH domains. Therefore, the detailed analysis of their structural features is reasonably needed. We calculated and analyzed the binding specificity of PH domains (receptors) for inositol phosphates (ligands). First, the geometries and electronic properties of the three inositol phosphates were calculated. The electronic properties and hydropathy of the receptors were investigated with structure-based analysis. Then, based on the structural characteristics of PH domains and inositol phosphates, the binding affinities and specificities of PH domains to inositol phosphates were analyzed.

### 3.1. Geometries and Electronic Properties of Inositol Phosphates

**Figure 3:**
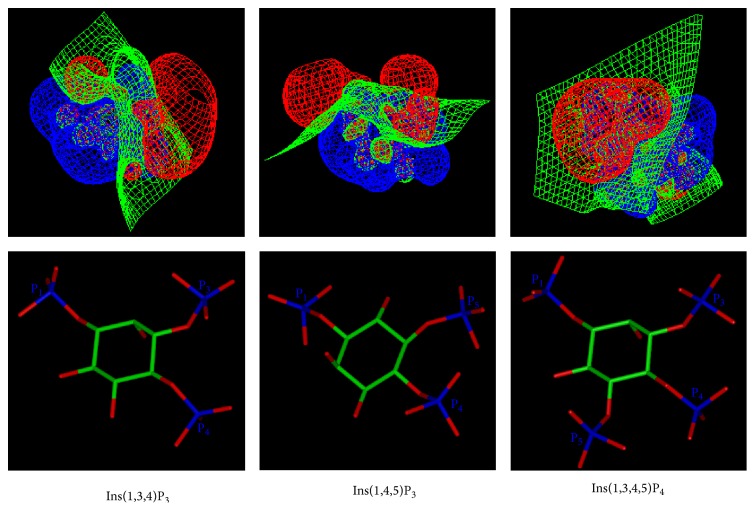
Comparison of the electrostatic potentials of three inositol phosphates. The contours of electrostatic potential at −5.0, 0.0, and 5.0 (kT/e) are coloured red, green, and blue, respectively.

The electrostatic potentials of the three inositol phosphates were calculated by QM (Figure 3). The calculated single point energies, geometries, and electronic properties are listed in Table 2. In all the optimized structures, the* myo*-inositol ring adopted the conformation with 1-axial/5-equatorial oxygen positions (C2-hydroxyl in axial position and the other hydroxyls/phosphates in the equatorial orientation).

The electronic charge distribution is apparently different for Ins(1,4,5)P_3_ and Ins(1,3,4)P_3_ (or Ins(1,3,4,5)P_4_) (Figure 3). In Ins(1,4,5)P_3_, the negative charge (in red) is concentrated on one side of the molecule. The molecules were oriented based on the superimposition of their inositol carbon atoms. To compare the geometries and properties of Ins(1,3,4)P_3_ and Ins(1,4,5)P_3_, the Ins(1,4,5)P_3_ was rotated by 180° to superimpose the phosphate groups of these two molecules. It has been reported that Ins(1,3,4,5)P_4_ is bound to Btk in the opposite orientation compared to Ins(1,4,5)P_3_ binding to Plc*δ* PH domain, although the interacting residues are in corresponding positions [3]. Figure 3 provides a qualitative explanation for the phenomenon, since the dipole moments of Ins(1,4,5)P_3_ and Ins(1,3,4,5)P_4_ point almost to the same direction when in inverted orientations.

The data in Table 2 confirmed the results of QM calculations. Although the chemical compositions of Ins(1,3,4)P_3_ and Ins(1,4,5)P_3_ are the same, the single point energies are different by 6.4 kcal/mol, indicating that Ins(1,4,5)P_3_ conformation is more theoretically stable. The comparison of the geometries of the compounds provides an explanation for the observation. The distances between the oxygen of 2-hydroxyl and the oxygen of 3-phosphate in Ins(1,3,4)P_3_ are 4.51, 3.89, and 2.64 Å, respectively, while the corresponding distances in Ins(1,4,5)P_3_ are 4.57, 4.26, and 2.66 Å, respectively. In Ins(1,3,4)P_3_, the axial 2-hydroxyl is near negatively charged vicinal equatorial 3-phosphate and thus the repulsion between them is greater than in Ins(1,4,5)P_3_. Both the dipole moment and the electronic spatial extent are greater in Ins(1,3,4)P_3_ than in Ins(1,4,5)P_3_.

The electronic spatial extent is a measure of molecular volume, whereas the dipole moment is an index of molecular polarizability. Ins(1,3,4)P_3_ thus has wider electronic charge distribution and greater polarity than Ins(1,4,5)P_3_. Since the electrostatic interaction is the main contributor for the interaction between PH domains and inositol phosphates. Ins(1,4,5)P_3_, which has smaller electronic spatial extent, binding to its preferred PH domain is weaker compared with Ins(1,3,4)P_3_. It is evident according to Figure 3 and Table 2 that the phosphate groups of Ins(1,4,5)P_3_ are on one side of the molecule and the shape is much flatter than that for Ins(1,3,4)P_3_. The different geometries and electronic properties between Ins(1,3,4)P_3_ and Ins(1,4,5)P_3_ contribute to the different specificities in PH domain interactions. The hydrophilic phosphate groups of inositol phosphates favour hydrophilic environment in the binding region of proteins. Among the three inositol phosphates, Ins(1,3,4,5)P_4_ requires the most hydrophilic environment for binding, due to having four hydrophilic phosphate groups. For Ins(1,4,5)P_3_, the binding environment in PH domain should be hydrophilic at one side of Ins(1,4,5)P_3_. The binding environment for Ins(1,3,4)P_3_ is less hydrophilic than that for Ins(1,4,5)P_3_, by virtue of the observation that the three phosphate groups in the molecule distribute separately and widely.

### 3.2. Electrostatic Properties and Hydropathy of PH Domains

**Figure 4:**
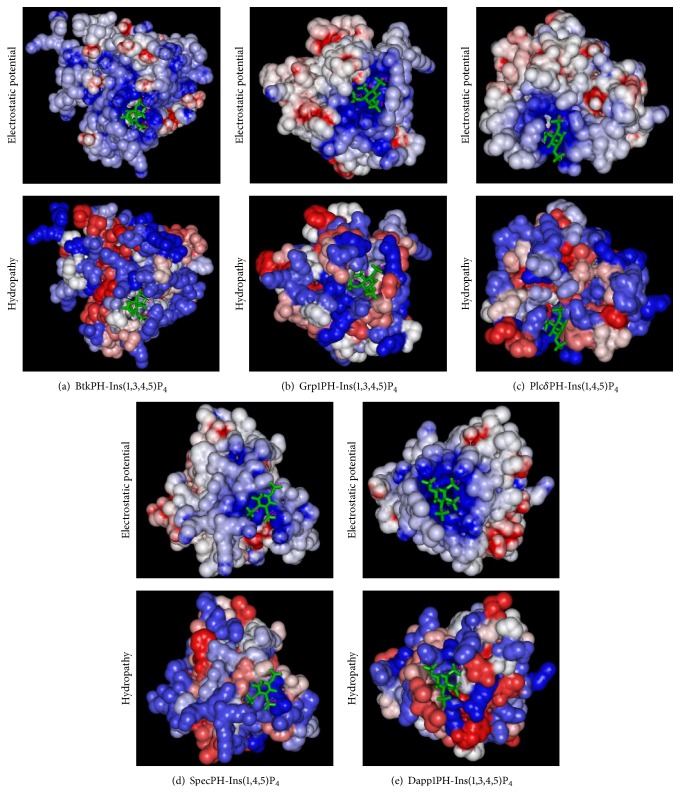
Electrostatic and hydrophobic surface representations of PH domains. Top: molecular surfaces coloured by electrostatic potential, from red (−10 kT/e) to blue (+10 kT/e). Bottom: Hydropathic surface representations, hydrophobicity, and hydrophilicity are coloured from red to blue.

The calculated electrostatic potentials of PH domain structures (Figure 4) indicate that the binding sites for inositol phosphates are conserved and positively charged and thus electrostatic interactions play the key role in the binding of inositol phosphates. The localization and orientation of the inositol phosphate binding sites can be estimated by calculating the electrostatic properties of PH domains. On the other hand, hydrophobicity is also important for both the function and the stability of a protein. Hydropathy profiles may indicate functional sites [38]. The hydropathy analyses of PH domains are shown in Figure 4. Hydropathy environments of the PH domain binding regions are not as conserved as the electronic charge distributions. Hydropathy profiles can help to explain the different affinities and specificities in inositol phosphate binding. The binding environments are most hydrophilic for PH domains in group 1 (Figures 4(a) and 4(b)). The hydrophilic binding environments for PH domains in group 2 are on one side of the bound Ins(1,4,5)P_3_ molecule (Figures 4(c) and 4(d)). The hydrophilic binding environments for PH domains in group 3 are less strict (Figure 4(e)). These observations are in agreement with the results obtained from the QM calculations of inositol phosphates.

We compared the structural profiles of electrostatic properties and hydropathy with results obtained by sequence profiles [24]. From both profiles, eight conserved extrema were found for structurally essential regions. For example, residues with smaller electronic charge correspond to the residues forming the hydrophobic core of PH domains, which may contribute to stabilizing the structure. According to the profiles of electronic charge concentration, the most charged segments are generally located in the *β*1/*β*2 and *β*7/*α*1 loops. Indeed, the *β*1/*β*2 loop is positively charged and thus appears to be the most important segment for the binding of inositol phosphates.

### 3.3. Binding Specificities of PH Domains

Table 3 lists the contact surface areas of phosphate groups in different PH domain-inositol phosphate complexes and the normalized complementarity (NC) function calculated by LIGIN program [34]. All the illegitimate contacts are of hydrophilic-hydrophobic type. In all cases, the C1-phosphate group has the lowest NC function indicating that the C1-phosphate group generally points outward and phosphoinositides can be replaced by inositol phosphates to study the binding specificities of PH domains. The binding affinities of inositol (tetra- and penta-) phosphates to Btk PH domain have the following order:

 Ins(1,3,4,5)P_4_ > Ins(1,3,4,5,6)P_5_ ≈ Ins(1,2,3,4,5,6)P_6_ > Ins(3,4,5,6)P_4_ > Ins(1,3,4,6)P_4_ > Ins(1,2,5,6)P_4_

By comparing with the experimental data of Btk PH domain [39], our computational results in Table 2 can be verified. In the structure of Btk PH domain-Ins(1,3,4,5)P_4_ complex, C2 and C6 hydroxyls point to neutral and hydrophobic environment, respectively. Therefore, C2-phosphate has minor effect on the binding to the Btk PH domain, while the phosphate at position 6 inhibits the complex formation. The binding environment of C1-phosphate (NC = 0.17) is less hydrophilic than that for C3, C4, and C5-phosphates (NC = 0.65, 0.76, and 0.57, resp.), and consequently the contribution of C1-phosphate to the binding affinity is smaller. Hydropathy allows the prediction of the order of affinities of compounds. Here, the binding orientations of inositol (tetra- and penta-) phosphates binding to the Btk PH domain were presumed to be the same, analogous to the binding to the Grp1 PH domain. The recent crystal structure of Btk domain with phosphatidylinositol further identified a key residue located at *β*1-*β*2 loop for the binding [40]. The side chain of residues in *β*1-*β*2 loop form hydrogen bonds with multiple diacylglycerol groups of PtdIns(3,4,5)P_3_. The binding specificities of *β*1-*β*2 loop are comparable with Ins(1,3,4,5)P_4_, which will be discussed in the next section.

In the structure of the Dapp1 PH domain-Ins(1,3,4,5)P_4_ complex the C6-hydroxyl is in a hydrophobic environment but it points outwards from the domain. It has only small effect on the affinity. The surroundings of C5-phosphate contain both hydrophobic and hydrophilic residues and thus C5-phosphate has minor effect on the interaction (NC = 0.20). In conclusion, the hydropathy analysis of the binding environment provides explanations for the experimentally obtained binding affinities as follows:

 Ins(1,3,4,5,6)P_5_  ≅ Ins(1,3,4,5)P_4_ > Ins(1,3,4,6)P_4_ > Ins(1,4,5,6)P_4_

Contrary to the Plc*δ* domain (Figure 4(c)), the *β*5/*β*6 loop in the spectrin PH domain (Figure 4(d)) is more hydrophilic and more positively charged than the *β*3/*β*4 loop. Consequently, the inositol phosphate binds between *β*1/*β*2 and *β*5/*β*6 loops in the spectrin PH domain.

### 3.4. Analysis of Binding Affinity

**Figure 5:**
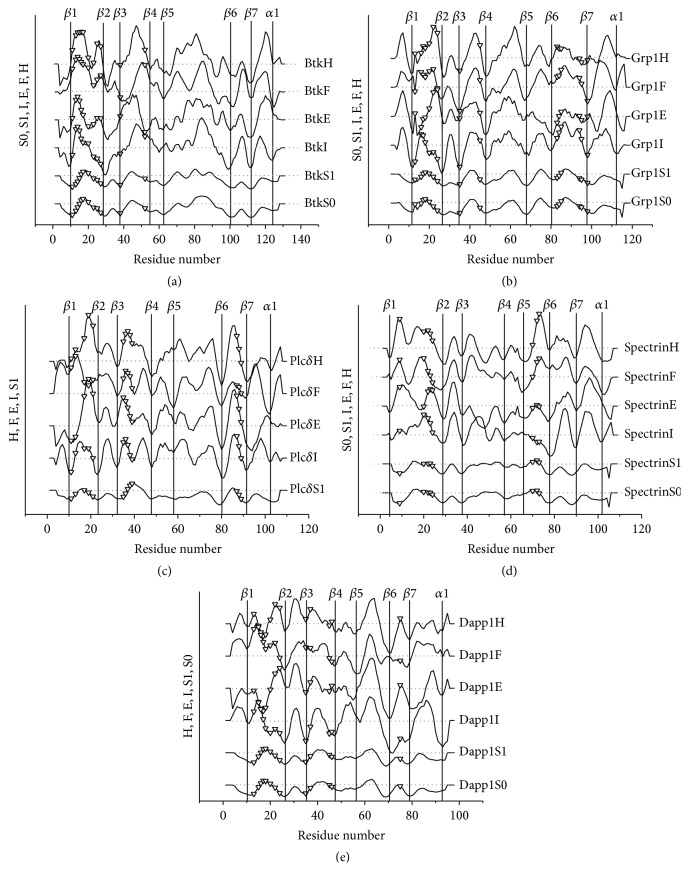
Ligand binding of the domains. The phosphatidyl inositol-binding residues are indicated by open triangles (∇). The insertions in the aligned sequences were deleted. The SASA for the PH domains and their complexes with inositol phosphates are marked with S0 and S1, respectively. H, F, E, and I represent hydropathy, flexibility, electronic charge concentration, and isotropic surface area, respectively.

The positions of binding sites in SASA profiles of known PH domain structures are shown in Figure 5. The PH domain binding sites are generally hydrophilic, flexible, and charged. The charge concentration, hydrophilicity, and flexibility are the main factors, which determine the binding affinity and specificity. Since the *β*1/*β*2 loop is located between the *β*3/*β*4 and *β*5/*β*6 loops, the residues in this loop play crucial roles in inositol phosphate binding. In Figures 5(a) and 5(b), the binding sites in the *β*1/*β*2 loop of Group 1 PH domains (Btk and Grp1) are located in the region of high hydrophilicity, high flexibility, and high electronic charge concentration. Group 1 PH domains are specific and have high affinity for Ins(1,3,4,5)P_4_. In addition, the Grp1 PH domain has high affinity to Ins(1,3,4,5)P_4_ including the contribution of the *β*6/*β*7 loop. In the complex of Btk PH domain-Ins(1,3,4,5)P_4_, the *β*6/*β*7 loop is not involved since it is hydrophobic.

**Figure 6:**
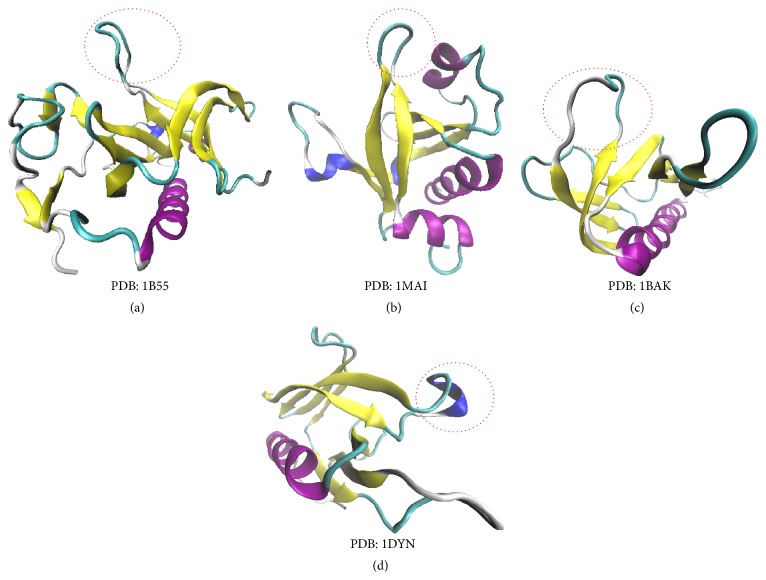
The structures of *β*1-*β*2 loops (red circles) in four PH domains.

The binding sites in the *β*1/*β*2 loop of Group 2 PH domains (Plc-*δ* and spectrin, Figures 5(c) and 5(d)) are also hydrophilic. The flexibility and electronic charge concentration are also high, but the electronic spatial extent and dipole moment of Ins(1,4,5)P_3_ are relatively small. The hydrophilic phosphate groups are distributed on one side of the molecule. Therefore their affinities are reduced compared to Group 1 and Group 3. Compared to Group 2 PH domains, the binding environment of Group 3 PH domain is less hydrophilic (Figure 5(e)), because the phosphate groups of their binding ligands are distributed separately, even for Ins(1,3,4)P_3_. The high binding affinity of the Dapp1 PH domain to Ins(1,3,4)P_3_ could be related to the electronic properties of Ins(1,3,4)P_3_. It can be seen that the Akt PH domain has similar profiles as Dapp1 [24, 41]. The Akt PH domain binds the Ins(1,3,4)P_3_ to the loops *β*1/*β*2, *β*3/*β*4, and *β*6/*β*7. As for Group 4 PH domains, it may be identified by inspecting the profile of electronic charge concentration and hydropathy. The peaks in loop *β*1/*β*2 are generally low and the binding of Group 4 PH domains for inositol phosphates is less specific. Accordingly, the positively charged *β*1-*β*2 loop in all the structures of PH domains appears to be the most important segment for the binding of inositol phosphates. The inositol-binding affinities can thus been explained by the length of *β*1-*β*2 loops. The *β*1-*β*2 loops of the BTK PH domain (Figure 6(a)) and Plc-*δ* PH domain (Figure 6(c)) contain 11 and 9 residues, showing higher inositol-binding affinities. In comparison, the *β*1-*β*2 loop of Akt PH domain (Figure 6(b)) and dynamin PH domain (Figure 6(d)) are shorter, and thus they have lower inositol-binding affinities.

**Figure 7:**
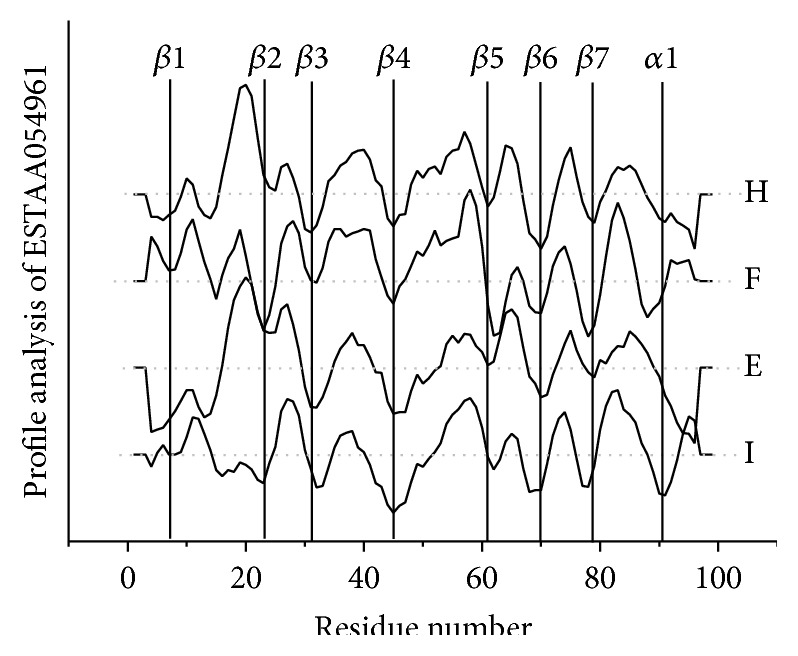
The profile analysis of ESTAA054961 PH domain.

For PH domains without structure, the sequence profiles can pinpoint possible binding sites, guide experiments, and provide understanding of the sequence-function relationships. A signature motif for 3-phosphate binding has been suggested [23]; however it does not distinguish between Group 1 and Group 3 PH domains. With profile analysis, these two groups are distinguished, since the binding sites of Group 1 PH domains are generally more hydrophilic. Motif information should be combined with the profile analysis to predict binding specificities of PH domains. By mapping the signature motif for 3-phosphate binding, Group 1 and Group 3 PH domains can be distinguished from Groups 2 and 4. Then Group 1 and Group 3 PH domains are partitioned by hydropathy profile analysis. By analyzing the hydropathy and electronic charge concentration, it is possible to identify Group 4 PH domains. Since Group 4 PH domains have less specificity and lower binding affinity, the hydrophilicity of the sequence profile is weaker and the electronic charge concentration of the sequence profile is lower.  Figure 7 gives an example of the prediction of the specificity of expressed sequence tag AA054961 PH domain, which bears the signature motif for 3-phosphate binding. Since the loop *β*1/*β*2 in this PH domain includes a notable hydrophobic peak, it is predicted to belong to Group 3.

## 4. Conclusions

**Figure 8:**
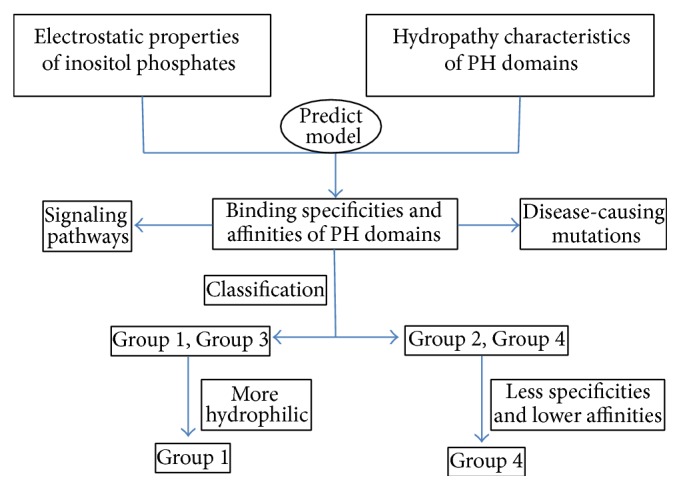
The key mode for the classification of PH domain based on inositol-binding specificity, which is helpful to the analysis of PH domains mediated signalling pathways and disease-causing mutations.

The different binding affinities and specificities of PH domains to the three inositol phosphates of Ins(1,3,4)P_3_, Ins(1,4,5)P_3_, and Ins(1,3,4,5)P_4_ were compared and explained from both the structural and sequence aspects. First, the electrostatics and geometric properties of the three inositol phosphates were calculated by a quantum mechanical method. Since the electronic charge distribution of the Ins(1,4,5)P_3_ is smaller, its interaction with PH domains is generally weak. The phosphate groups in the Ins(1,4,5)P_3_ are on one side of the molecule and the binding region is more hydrophilic on one side of the binding molecule than for Ins(1,3,4)P_3_. Then, the structure-based electrostatic properties and hydropathy of PH domains profiles showed that hydrophobic environment is essential for the binding specificity. These structural results are compared with sequence profiles for the analysis of binding specificity of PH domains, which also proved the essential role of hydrophobic environment for the binding specificity. The agreement of information from 1-dimensional sequence profiles and 3-dimensional structures provides a simple but practical method to investigate sequence-structure relationship of PH domains. The overall flowchart of our research is summarized in Figure 8, which also contain two future directions.

PH domains can also be specifically identified and combined with signalling molecules, such as PTEN and PI3K [8]. It constitutes the basis for PH domains to participate in a variety of signalling pathways. Therefore, further understanding of the interaction between inositol phosphates and its downstream molecules not only reveals a consistent picture of PH domain-mediated signal network system but also provides new insights into the mechanism of diseases with respect to these signalling pathways. Although MD simulation has been used to understand the interaction of PKB PH domain with inositol phosphates involved in the PI3K pathway [42], it remains a major challenge in the field.

In our previous work, the relationship between sequence profiles of binding sites and the effect of disease-causing PH domain mutation was analyzed, and MD method has been used to classify “folding mutation” and “disease-causing mutation” [25]. However, the analysis and discussion of disease-causing variations affecting binding specificities and affinities pose another challenge. For example, Btk PH domain is the most studied PH domain which contains the highest number of unique disease-causing variations among the human protein kinases. The PON-BTK provides [43] a method for analyzing and classifying disease-causing mutations. With this mutation data, it is possible to reveal the basis of XLA by binding analysis. We hope that, in the new future, our method would be applied to PH domains to understand the basis of diseases with respect to inositol phosphates involving signalling pathways and harmful mutations for PH domains.

## Figures and Tables

**Table 1:** Binding specificities of representative PH domains.

	Group 1	Group 2	Group 3	Group 4
PI(3,4,5)P_3_/Ins(1,3,4,5)P_4_	+^a^	+	+	−
PI(4,5)P_2_/Ins(1,4,5)P_3_	−	+	−	−
PI(3,4)P_2_/Ins(1,3,4)P_3_	−	−	+	−
PH domain	Btk, Grp1, Gap1^m^ Gap1^IP4BP^, Vav, cytohesin-1, Sos, ARNO, TIAM1-N	PLC*δ*1, *β*Ark, *β*-spectrin, DAGK*δ*, RasGAp, OSBP, IRS-1, Plec-N	Dapp1, Akt, PDK1	Dynamin, TIAM1-C

^a^+, − represent specific binding or nonspecific binding. Gap1^IP4BP^ represents one of the members of Ras GTPase-activating proteins and Gap1^m^ represents the mammalian counterpart of the *Drosophila* Gap1 gene.

**Table 2:** Comparison of the geometries and electronic properties of myo-inositol phosphates.

	Ins(1,3,4)P_3_	Ins(1,4,5)P_3_	Ins(1,3,4,5)P_4_
Distance (O_2_-O_P_3__, Å)^a^	4.51; 3.89; 2.64	—	4.55; 3.40; 2.72
Distance (O_6_-O_P_5__, Å)^b^	—	4.57; 4.26; 2.66	4.62; 3.91; 2.84
Electronic spatial extent (Å^2^)	10026	9868	13820
Dipole moment (Debye)	17.2	11.7	10.6
Energy (HF) (a.u.)	−2369.20	−2369.21	−2932.45
Energy (MP2) (a.u.)	−2371.76	−2371.77	−2951.77
ΔEnergy (MP2) (kcal/mol)^c^	6.4	0.0	—

^a^Distances between oxygen atom of 2-OH and three oxygen atoms of 3-PO_3_ in Ins(1,3,4)P_3_ or Ins(1,3,4,5)P_4_.

^b^Distances between oxygen atom of 6-OH and three oxygen atoms of 5-PO_3_ in Ins(1,4,5)P_3_ or Ins(1,3,4,5)P_4_.

There is no result for O_2_-O_P_3__ distances in Ins(1,4,5)P_3_ and O_6_-O_P_5__ distance in Ins(1,3,4)P_3_, because they do not contain 3-PO_3_ and 5-PO_3_ groups, respectively.

^c^The energy difference is calculated only for Ins(1,3,4)P_3_ and Ins(1,4,5)P_3_, because they belong to the same molecule but different conformations.

**Table 3:** Comparison of ligand-protein contacts in 5 PH domain-inositol phosphate complexes.

		Contact surface area (Å^2^)			Normalized complementarities
		Legitimate contacts	Illegitimate contacts	Complementarities	
1B55	P_1_-O_3_	31.3	10.6	20.7	0.17
	P_3_-O_3_	85.0	16.0	69.0	0.65
	P_4_-O_3_	86.8	17.9	68.9	0.76
	P_5_-O_3_	81.6	19.7	61.9	0.57

1FGY	P_1_-O_3_	31.0	23.8	7.2	0.06
	P_3_-O_3_	89.6	21.8	67.8	0.61
	P_4_-O_3_	114.8	8.5	106.3	1.00
	P_5_-O_3_	104.5	11.1	93.4	0.80

1MAI	P_1_-O_3_	44.0	0.5	43.5	0.34
	P_4_-O_3_	83.0	17.8	65.2	0.55
	P_5_-O_3_	85.8	0.3	85.5	0.72

1BTN	P_1_-O_3_	33.6	0	33.6	0.27
	P_4_-O_3_	69.4	23.5	45.9	0.43
	P_5_-O_3_	72.6	0	72.6	0.68

1FAO	P_1_-O_3_	34.8	18.1	16.7	0.13
	P_3_-O_3_	82.9	23.1	59.8	0.55
	P_4_-O_3_	102.1	3.1	99.0	0.98
	P_5_-O_3_	45.0	20.9	24.1	0.20
